# Studying human-AI collaboration protocols: the case of the Kasparov’s law in radiological double reading

**DOI:** 10.1007/s13755-021-00138-8

**Published:** 2021-02-05

**Authors:** Federico Cabitza, Andrea Campagner, Luca Maria Sconfienza

**Affiliations:** 1 Università degli Studi di Milano-Bicocca, Viale Sarca 336, 20126 Milan, Italy; 2 Department of Biomedical Sciences for Health, University of Milan, Milan, Italy; 3 IRCCS Istituto Ortopedico Galeazzi, Milan, Italy

**Keywords:** Kasparov’s Law, Interaction protocols, Double reading, Collective intelligence, Hybrid intelligence

## Abstract

**Purpose:**

The integration of Artificial Intelligence into medical practices has recently been advocated for the promise to bring increased efficiency and effectiveness to these practices. Nonetheless, little research has so far been aimed at understanding the best human-AI interaction protocols in collaborative tasks, even in currently more viable settings, like independent double-reading screening tasks.

**Methods:**

To this aim, we report about a retrospective case–control study, involving 12 board-certified radiologists, in the detection of knee lesions by means of Magnetic Resonance Imaging, in which we simulated the serial combination of two Deep Learning models with humans in eight double-reading protocols. Inspired by the so-called Kasparov’s Laws, we investigate whether the combination of humans and AI models could achieve better performance than AI models alone, and whether weak reader, when supported by fit-for-use interaction protocols, could out-perform stronger readers.

**Results:**

We discuss two main findings: groups of humans who perform significantly worse than a state-of-the-art AI can significantly outperform it if their judgements are aggregated by majority voting (in concordance with the first part of the Kasparov’s law); small ensembles of significantly weaker readers can significantly outperform teams of stronger readers, supported by the same computational tool, when the judgments of the former ones are combined within “fit-for-use” protocols (in concordance with the second part of the Kasparov’s law).

**Conclusion:**

Our study shows that good interaction protocols can guarantee improved decision performance that easily surpasses the performance of individual agents, even of realistic super-human AI systems. This finding highlights the importance of focusing on how to guarantee better co-operation within human-AI teams, so to enable safer and more human sustainable care practices.

## Introduction

The integration of computational systems endowed with some form of Artificial Intelligence (AI) into medical practices is advocated for several reasons, the main ones being the promises to bring more efficiency (e.g., [21]) and effectiveness to those practices. Although efficiency and effectiveness are very broad concepts, we intend efficiency gains in terms of any process improvement for which the throughput of medical services is improved and wait times reduced, or for which their provision is guaranteed at minor costs and less resource consumption; and effectiveness gains as any improvement in diagnostic accuracy (fewer errors), safety (e.g., fewer adverse events, infections, re-admissions), and better outcomes and satisfaction.

Moreover, these two broad dimensions are often connected in multiple ways. For instance in case of double-reading processes for mammography screening, the accuracy of an AI used to provide a rapid second opinion was non-inferior to serial reading by two radiologists (c.f., effectiveness), and the simulated workload of the second reader was reduced by 88% (cf. efficiency) [28]. In case of Magnetic Resonance Imaging (MRI), AI-based image reconstruction and postprocessing methods can reduce scan times, to 10-minutes sessions or even less, while maintaining equivalent image quality [50]. This would not only lead to higher patient satisfaction, especially in case of claustrophobic people, but also allow hospitals to double or triple the number of MRI tests that can be performed each day, also by making MRI a “walk-in service” for emergency triage (like X-rays or CT).^1^ The same techniques of deep learning can yield higher-quality images on scanners with lower field strengths (thus indirectly improving diagnostic accuracy in many healthcare facilities), as well as it can enable the use of lower (or even no) doses of gadolinium-based contrast agents, so that safety could be improved by reducing the exam toxicity and the odds of adverse reactions [17]. On the other hand, such a disruptive increase in exam throughput would also require radiologists and specialists read and report more scans, or hospitals to hire more of these specialists, thus eroding potential efficiency gains [47].

This can promote a further business case of medical AI in diagnostic imaging, that is the use of AI assistants as decision support: this case is usually seen from two opposing stances: the perspective of the cognitive augmentation of the reading radiologist; or the opposite stance that contrasts human radiologists and these systems in virtue of their equal, if not superior, accuracy [30]. However, also a third perspective can be adopted: the one that recognizes the necessity of this alliance in diagnostic tasks for the sustainability of the healthcare systems that leverage AI for more robust business cases aimed at gaining efficiency and safety improvements first.

In this paper, we will focus on this third perspective: namely, *how to integrate human readers and AI systems together*. One of the most interesting contributions to the comprehension of the dynamics that can characterize human–machine teams, although still neglected by academic research, was proposed by Garry Kasparov in [26]. His position was first presented in an influential 2014 book by Brynjolfsson and McAfee [5] and it is often summarized in terms of the so-called *Kasparov’s law*, and rendered in the following schematic and composite way  [26][p. 236]:

1. Weak Human + Machine + Better Process > Strong Machine;
2. Weak Human + Machine + Better Process > Strong Human + Machine + Inferior Process

where the inequality sign can have different but related meanings, like “is superior to” to some respect, or “beats” (in some game, like in free-style chess, where any arrangement of humans and computers are allowed), or just “is better than” according to some quality criterion.

Proving the above law is an exercise of low utility (if reasonable at all), also because this expression regards two apparent conjectures about the nature of human-AI collaboration, not a general principle: the application of this “law” depends on aspects like the nature of the tasks at hand, differences between the humans involved (weak vs strong), and what being a superior and inferior process actually means. These elements would make any demonstration nothing more than a local curiosity lacking any ambition of being replicable and transferable to other settings, let alone application domains.

However, in this formulation we find interesting the concept of *process*, which we interpret in terms of *protocol* stipulating how human decision makers should interact with the machines that support them. Our aim is to to see if, among some human-AI interaction protocols that can be conceived for radiological double reading, there is some configuration for which the Kasparov’s law applies, and therefore it is suggestive of some practically significant difference between processes with respect to some dimension of interest.

Various *interaction protocols* can be designed depending on the complexity of the decision task, its typical load and its requirements: processes can range from those that do not provide for direct collaboration (like voting) to protocols that, conversely, enable and support a rich exchange where humans might even engage with machines as if they were teammates [42], so that the so-called *hybrid intelligence* [2] can emerge, i.e., a specialization of the more common concept of *collective intelligence*, where AI machines are members of the collective. For this reason, this study is also a contribution to the ongoing multidisciplinary research investigating cases of *collective intelligence* in medicine, that is on how to design viable and reliable methods by which groups of agents can achieve a better performance, in tasks that would usually require some intelligence, knowledge and competence, than single agents (including medical decision making, e.g. [27, 38, 49]) by combining their multiple contributions together, regardless of their human or “machinic” nature (e.g. [22, 48]).

However, still little research has so far aimed at understanding what the best *human-AI interaction protocols* in collaborative medical tasks are, even in settings that are more viable for the current state of the art in medical AI, like independent double-reading screening tasks [28].

To fill the above mentioned literature gap, and inspired by the mentioned Kasparov’s Law, in this paper we will report about a retrospective case–control study in the detection of knee lesions by means of Magnetic Resonance Imaging (MRI) in which we simulated the serial combination of an AI in a number of *double-reading* protocols. Our research questions are: do specific human-AI *interaction protocols* exist by which, on one hand, combining humans and AI together can achieve significantly better performance than AI alone and, on the other hand, weaker readers can out-perform stronger readers even if they use the same computational support? If we show that such protocols exist, the intuition behind the Kasparov’s law would be proved sound and the its implications would deserve a dedicated line of research.

As mentioned above, in what follows we will focus on the case of *double reading*, in which two or more radiologists, often called *observers* or *readers*, consider the same clinical case by reading the same images [16]. This general scheme applies to different practices (see [16] for a review of these variations); more in particular, we will focus on *double reading with pseudo arbitration*: this is the (diagnostic) deliberation method by which two observers considers a case, serially and independently of each other to avoid undue and mutual influence (mainly in terms of priming, framing, and social desirability bias), and a third observer does the same if the two former interpretations (in terms of normal-health / abnormal-pathological exam) differ (i.e., when a conflict occurs). In pseudo-arbitration also the third observer (sometimes called *arbiter*) considers the case unaware of the previous disagreements, to avoid undue influence (like priming and groupthink effects) and the final decision (diagnosis) is taken by majority voting.

## Methods

**Fig. 1:**
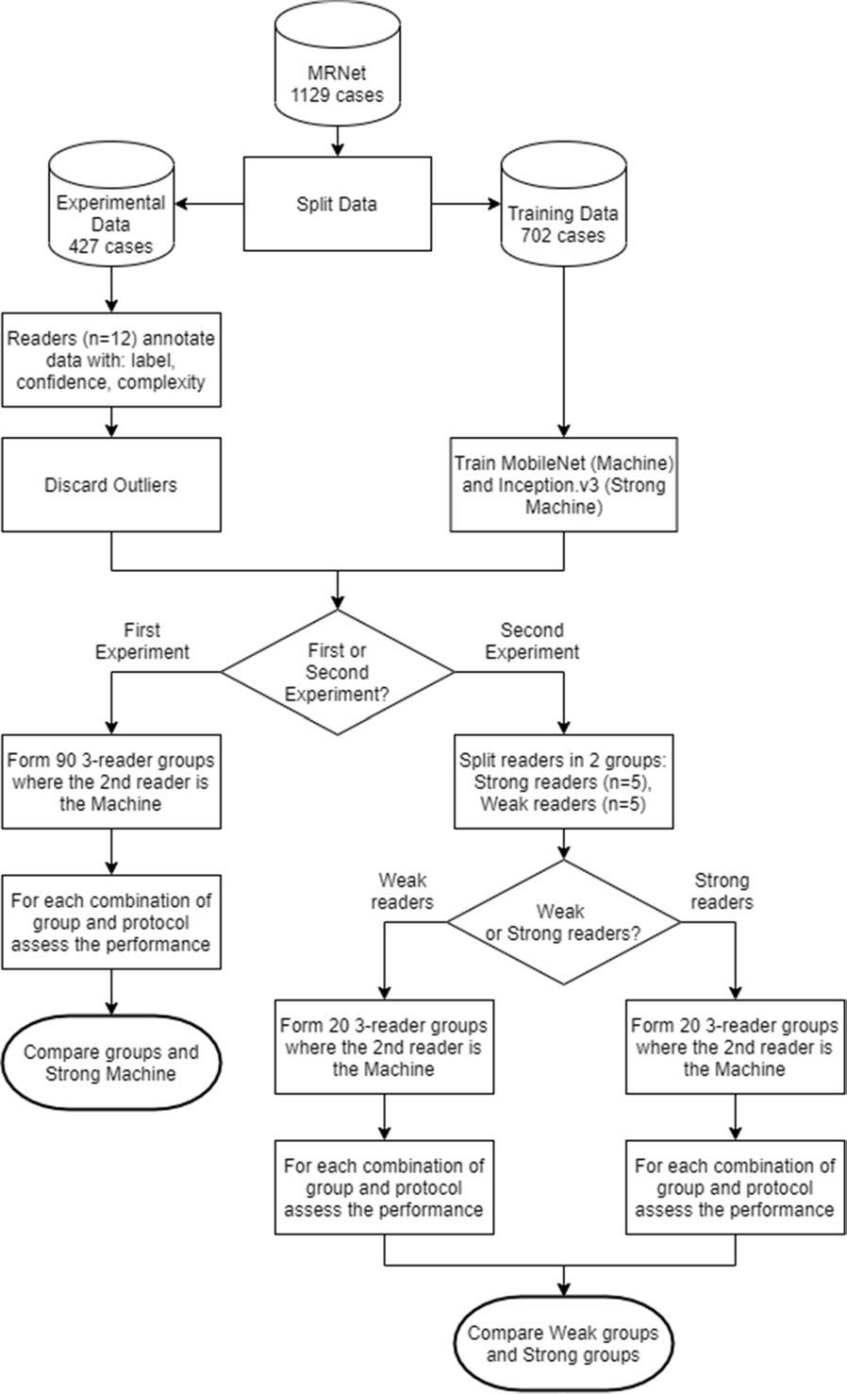
Flowchart representation of the adopted experimental methodology

In this Section we describe the experimental methodology adopted to study the previously mentioned research question: A graphical representation of the experimental workflow is reported in Fig. 1.

To implement the double-reading (with pseudo-arbitration) setting for our study, we involved 12 board-certified specialized radiologists, from several orthopedic centers in Italy, but mainly from the IRCCS Istituto Ortopedico Galeazzi of Milan, in a retrospective case–control study in the detection of knee lesions (i.e., meniscus and ligament tears) by means of Magnetic Resonance Imaging (MRI). The data collection was performed through an online questionnaire platform (Limesurvey, version 3.188) and the radiologists were invited to participate in the study by personal email. Each of the 12 radiologists was asked to annotate a collection of 427 MRI images (randomly sampled from the Stanford MRNet image repository, which contains a total of 1129 cases) so to have a balanced dataset with respect to abnormal and normal cases. The radiologists were also asked to report, for each case, the subjectively perceived complexity of the given case, and their subjective confidence in the provided diagnosis. Specifically, both case complexity and confidence were represented as values on a discrete ordinal scale with, respectively, 4 and 5 values. The ordinal levels were subsequently normalized to be represented on a [0, 1] numerical scale.

**Fig. 2:**
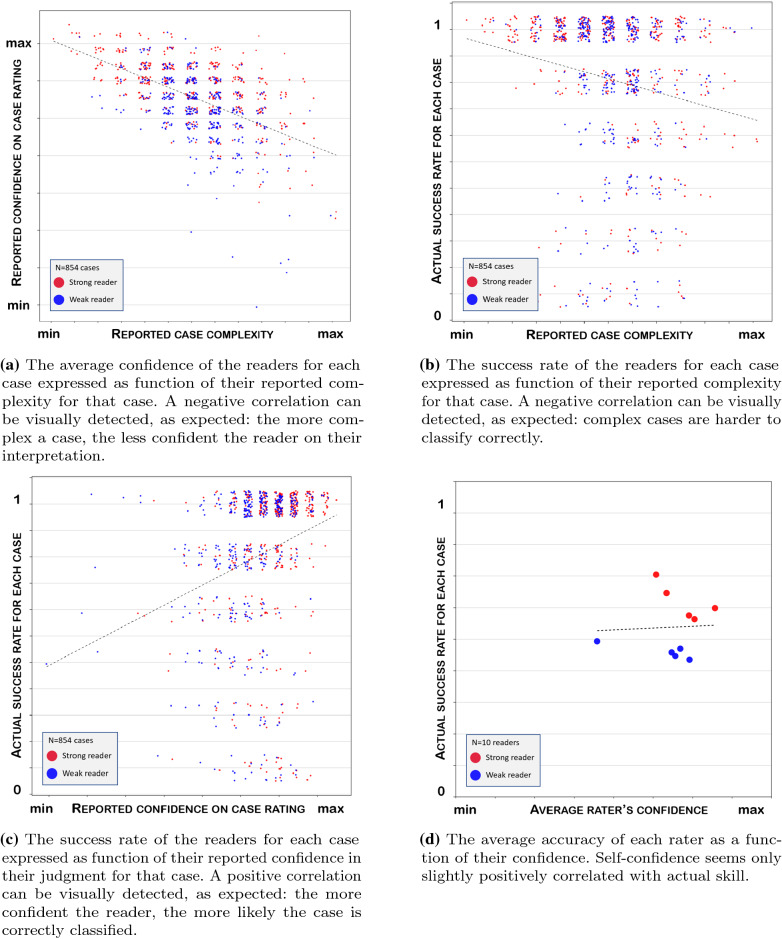
Scatterplot of the associations between accuracy and the other dataset metadata. Red circles indicate the “strong” readers, the blue circle the “weak” ones.

See Fig. 2a–d, to see how average (reader-wise) reported confidence and average reported complexity correlate with case-wise success rate by the 10 readers.

Each radiologist was evaluated in terms of accuracy with respect to the MRNet gold standard: the average accuracy was $80.96\% \pm 1.4\%$ (95% confidence interval). The most accurate (86.18%) and least accurate (77.52%) observers were discarded as outliers, thus in the subsequent analysis we considered the group of 10 remaining radiologists (M = $80.77\%$, min = $78.69\%$, max = $84.07\%$, see Table 1).

**Table 1:** Accuracy of the 10 radiologists involved in the experiments.

Reader	1	2	3	4	5	6	7	8	9	10
Acc.	81.26	79.39	81.5	78.69	79.16	84.07	79.86	81.97	78.92	82.9

After the selection of the 427 cases given to the human readers and the collection of the readers’ annotations, the remaining 702 cases (for a total of 24553 training images) were subsequently used to train two Convolutional Neural Network models: a MobileNet [24] model (88 layers) and an InceptionV3 [46] model (159 layers). The models were then evaluated on the 427 MRI images used in our study reporting $81.72\%$ and $84.54\%$ accuracy, respectively.

**Fig. 3:**
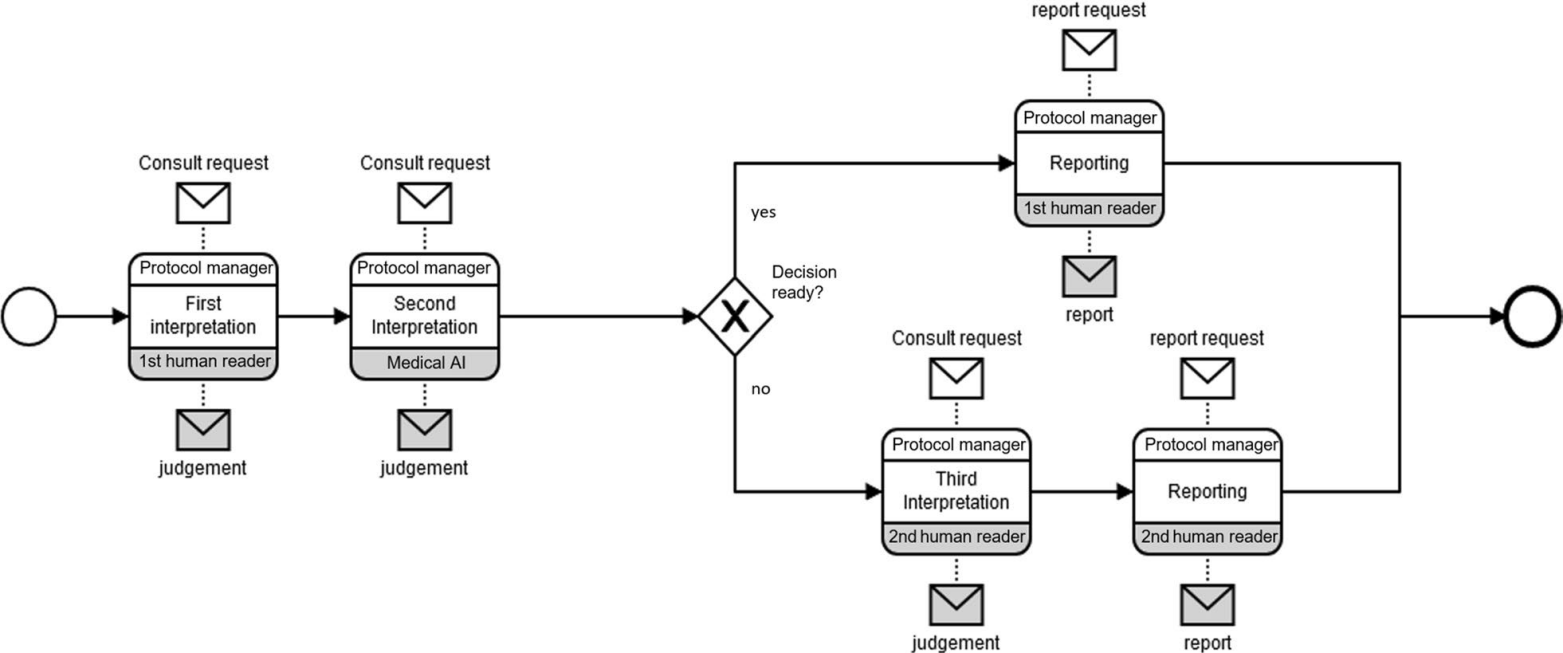
A choreography diagram describing the general articulation of the image reading and reporting tasks. We recall this kind of diagram only represents interactions, no data processing. The articulation depicted above reflects the assumption that radiological reports will be written and signed by human radiologists for a long time yet, mainly due to legal liability concerns [37] (even if systems that are capable of generating meaningful reports linked to a diagnostic classification have already been described, e.g. [15]).

For the evaluation of the Kasparov’s Laws we considered human-AI groups, employing 8 different double-reading protocols, which are all variations of the general interaction protocol depicted in Fig. 3.

1. *Simple-Majority Protocol*: The first and second observer provide their judgments, the third observer is involved if and only if the first two observers disagree. The final decision is the majority choice of the three observers in the team;
2. *Accuracy-Weighted Majority Protocol*: As in the simple-majority protocol, but the judgment produced by each observer is weighted by their accuracy;
3. *Confidence-Weighted Majority Protocol*: As in the simple-majority protocol, but the judgement provided by each observer is weighted by the confidence of the respective observer on the given case;
4. *Specificity-oriented Protocol*: The first observer provides their judgment, the second observer is involved if and only if the first observer deemed the case abnormal. If the first two observers disagree, then the third observer is also involved. The result is the majority vote of the involved observers;
5. *Sensitivity-oriented Protocol*: The first observer provides their judgement, the second observer is involved if and only if the first observer deemed the case normal. If the first two observers disagree, then the third observer is also involved. The final decision is the majority choice of the involved observers;
6. *Cautious Protocol*: The first two observers provide their judgments, their subjective confidences $conf_1, conf_2$ and perceived case complexity scores $compl_1, compl_2$. If $|conf_1 - conf_2| \ge \tau$ and $\frac{compl_1 + compl_2}{2} \le \delta$, where $\tau , \delta$ are two thresholds in [0, 1]; then the team’s decision is the same as the one provided by the observer with greater confidence. Otherwise, the third observer is involved and the result is the majority choice of the involved observers;
7. *Presumptuous Protocol*: The first two observers provide their judgements and their subjective confidence scores $conf_1, conf_2$. The team’s decision is the same as the one provided by the observer with greater confidence. If $conf_1 = conf_2$, then the third observer is involved and the result is the majority choice of the involved observers;
8. *OR Rule*: The first observer provides their judgment, the second observer is involved if and only if the interpretation of the first observer is normal and, in that case, the decision of the team is the same as the second observer’s.

**Fig. 4:**
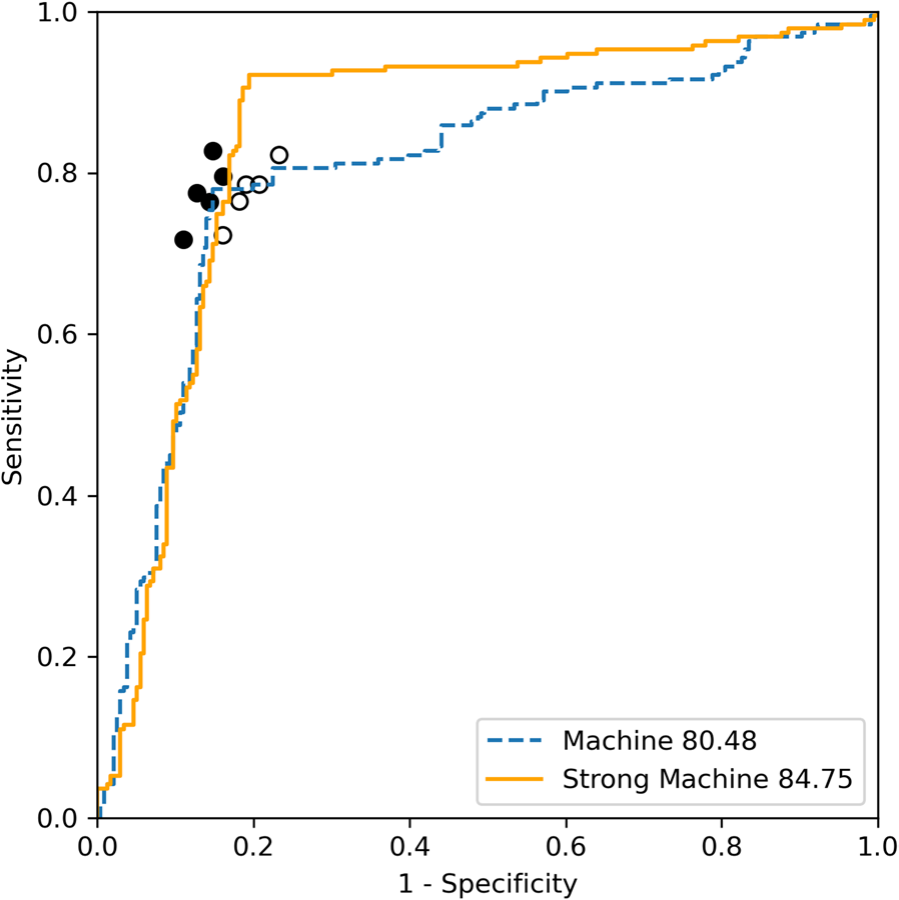
Performance of the 10 observers and the two AI models, represented in ROC space. Empty circles correspond to the weak image readers, full circles to the strong ones involved in the study.

Since the Kasparov’s Law (KL), as mentioned in the Introduction, can be framed as two different statements, we designed two different experiments:

1. In order to evaluate the first KL statement, we used the InceptionV3 model as the “Strong” machine (which was actually more accurate than the best human reader involved in this study), and we then used the MobileNet model as the Machine (second reader) to be used in the “better” process of human-AI collaboration. Specifically, the Machine was always recruited as the second observer, like done in [39], where it was found that combining the first reader with the best algorithm identified more pathological cases than having a human as the second reader. In doing so, we considered a total of 90 3-reader 2-permutations.^2^ For each group we evaluated the performance of each of the 8 double reading protocols;
2. In order to evaluate the second KL statement, we split the observers in two groups: the group of *weak* observers, that is the 5 least accurate observers (accuracy from 78.69% to 79.86%, M = 79.20, SD = 0.46), and the group of *strong* observers, that is the 5 most accurate observers (accuracy from 81.26% to 84.07%, M = 82.34, SD = 1.15). The weak observers were, on average, significantly less accurate than the strong one (t = 5.66, df = 8, p = 0.002). The performance of the observers and the ROC curves of the AI models are reported in Fig. 4. As for the first experiment, we used the MobileNet model as the Machine (second reader): thus, we considered 20 2-permutations of weak readers, and 20 2-permutations of strong readers. For both groups of observers we evaluated the performance of all 8 considered protocols.

For both experiments, for the Cautious and Presumptuous protocols, the values of $\tau$ and $\delta$ were set as:

1 $$\begin{aligned} \tau&= \frac{1}{427}\sum _{c \text { case}}\left( {\begin{array}{c}10\\ 2\end{array}}\right) ^{-1}\sum _{o_1, o_2 \text { observers}} |conf_1 - conf_2|\end{aligned}$$

2 $$\begin{aligned} \delta&= \frac{1}{427}\sum _{c \text { case}}\frac{1}{10}\sum _{o_i \text { observer}} compl_i \end{aligned}$$

that is, as, respectively, the mean of differences in the reported confidences, and the mean reported case complexity. Thus, the values of $\tau$ and $\delta$ were set, respectively as $\tau = 0.17$ and $\delta = 0.70$.

After collecting the annotations produced by each of the considered groups (for each protocol), we then evaluated each of the groups (and protocol), for both experiments, in terms of six different evaluation metrics, defined as follows:

- $$accuracy = \frac{\text {number of correct cases}}{\text {total number of cases}}$$;
- $$sensitivity = \frac{\text {number of true positives}}{\text {total number of positive cases}}$$;
- $$specificity = \frac{\text {number of true negatives}}{\text {total number of negative cases}}$$;
- $$efficiency = \frac{\text {number of correct cases}}{\text {total number of single judgments}}$$;
- the 1-order harmonic mean of *sensitivity* and *specificity*, that is $\frac{2}{\text {sensitivity}^{-1} + \text {specificity}^{-1}}$;
- the 2-order harmonic mean of *accuracy* and *efficiency* (henceforth, $F_2(acc, eff)$), that is $(1+2^{2})*\frac{\text {accuracy}*\text {efficiency}}{\text {accuracy}+2^2*\text {efficiency}}$; 3

As regard the first experiment, we evaluated each group and then compared the strong machine against the average group performance, in terms of accuracy, sensitivity, specificity and the harmonic mean of the latter two metrics. We also report the efficiency of each protocol. As regard the second experiment, we evaluated each weak and strong group and then compared the average group performance for each of the two samples, in terms of all the above described metrics.

In order to assess the presence, if any, of statistically significant differences we applied statistical hypothesis testing methods: specifically, as regards the first experiment we applied the *one sample T test* comparing, for each considered metrics and protocol, the results of the 90 groups against the results of the Strong Machine (that is, we tested the hypothesis that the results of the groups were drawn from a distribution with average equal to the results of the Strong Machine); while as regards the second experiment, for each metric and protocol, we applied the non-parametric Kolmogorov-Smirnov test to compare the distributions of the weak and strong groups. In both cases, in order to control for multiple comparisons, we applied the Bonferroni correction to the obtained p-values: significance was assessed, as standard, comparing the corrected p-values against a 95% confidence level (that is, $\alpha = 0.05$).

## Results

In what follows, we report the findings observed in both the experiments designed as in “Methods” section, covering a statement in the Kasparov’s Law each.

### First statement of the Kasparov’s Law

**Fig. 5:**
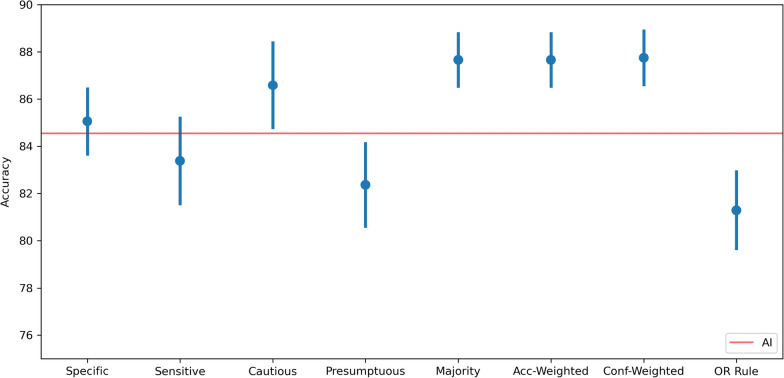
Average group accuracy and 95% confidence intervals for each of the 8 different protocols. The red line represents the accuracy of the strong machine.

**Fig. 6:**
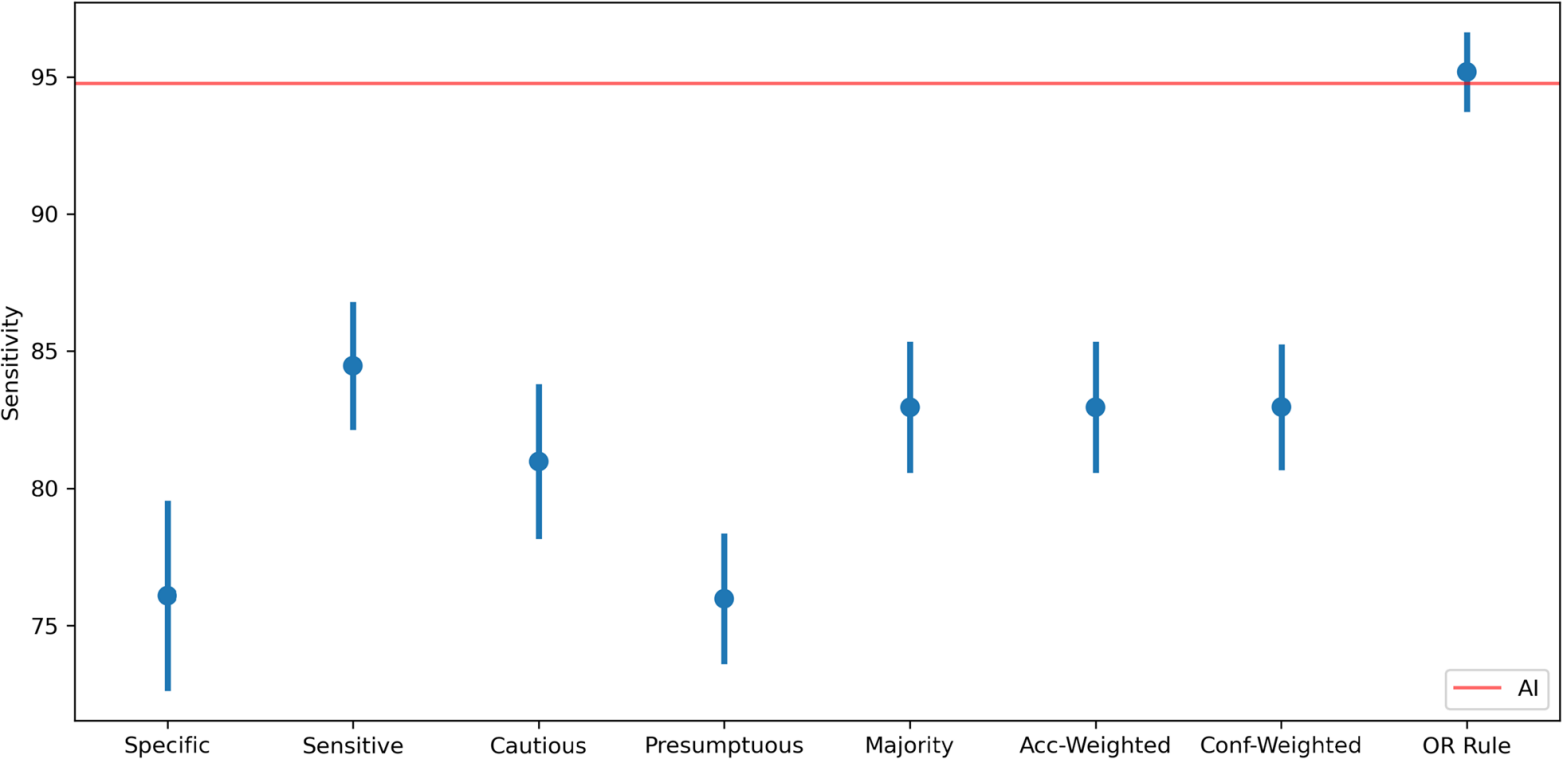
Average group sensitivity and 95% confidence intervals for each of the 8 different protocols. The red line represents the sensitivity of the strong machine.

**Fig. 7:**
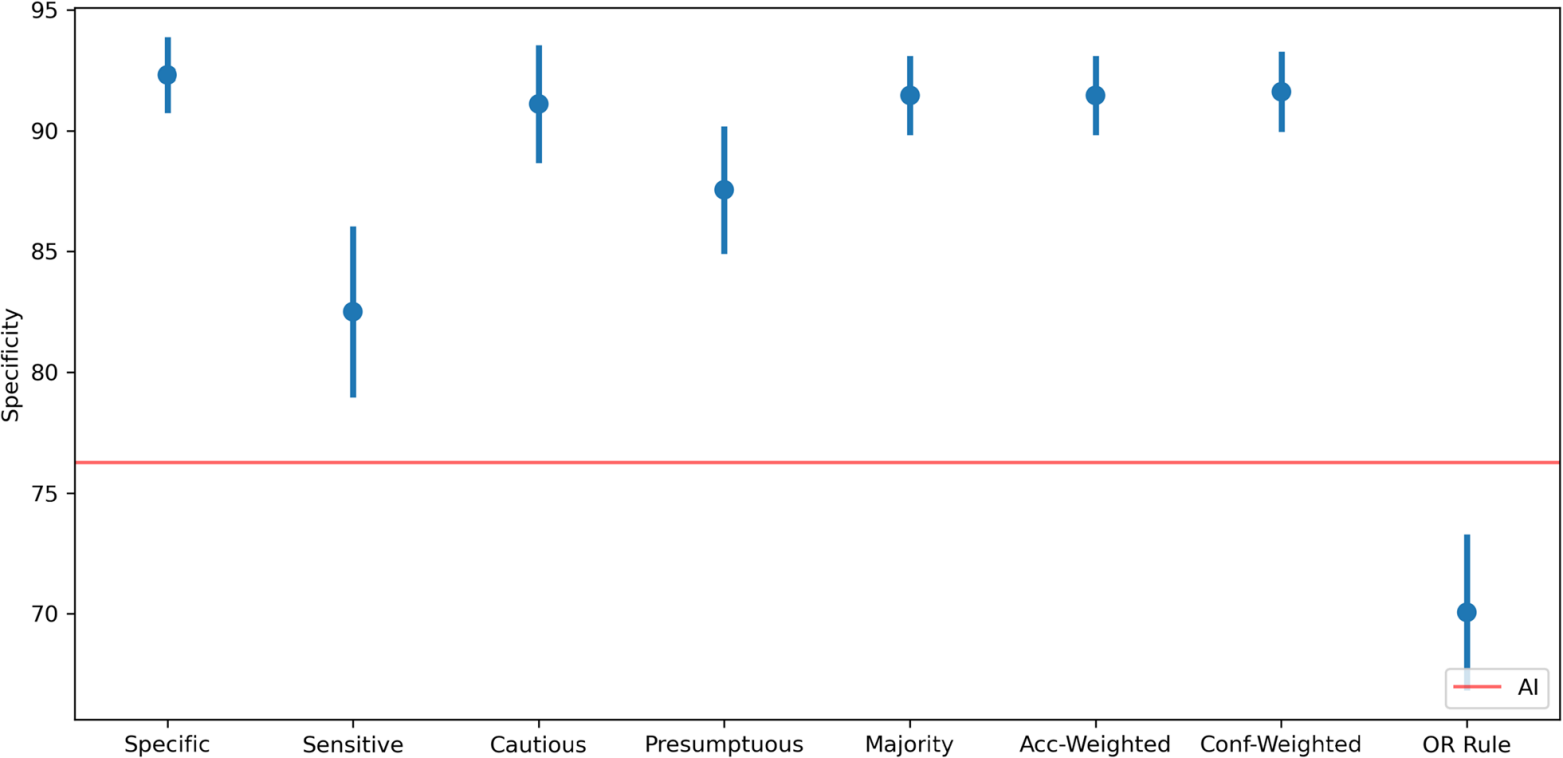
Average group specificity and 95% confidence intervals for each of the 8 different protocols. The red line represents the specificity of the strong machine.

**Fig. 8:**
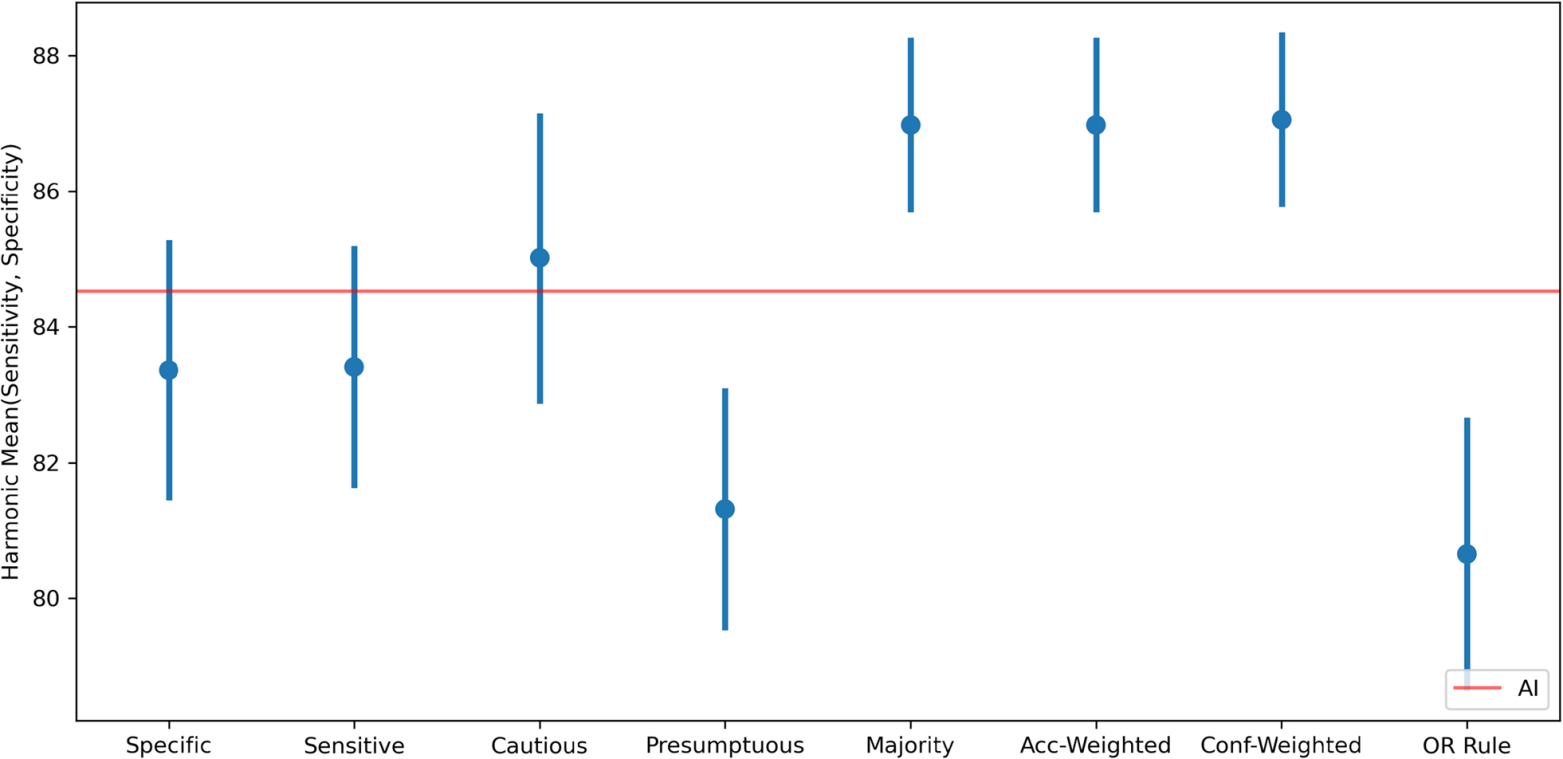
Average group value of the harmonic mean of sensitivity and specificity, along with 95% confidence intervals for each of the 8 different protocols. The red line represents the harmonic mean (of sensitivity and specificity) of the strong machine.

**Fig. 9:**
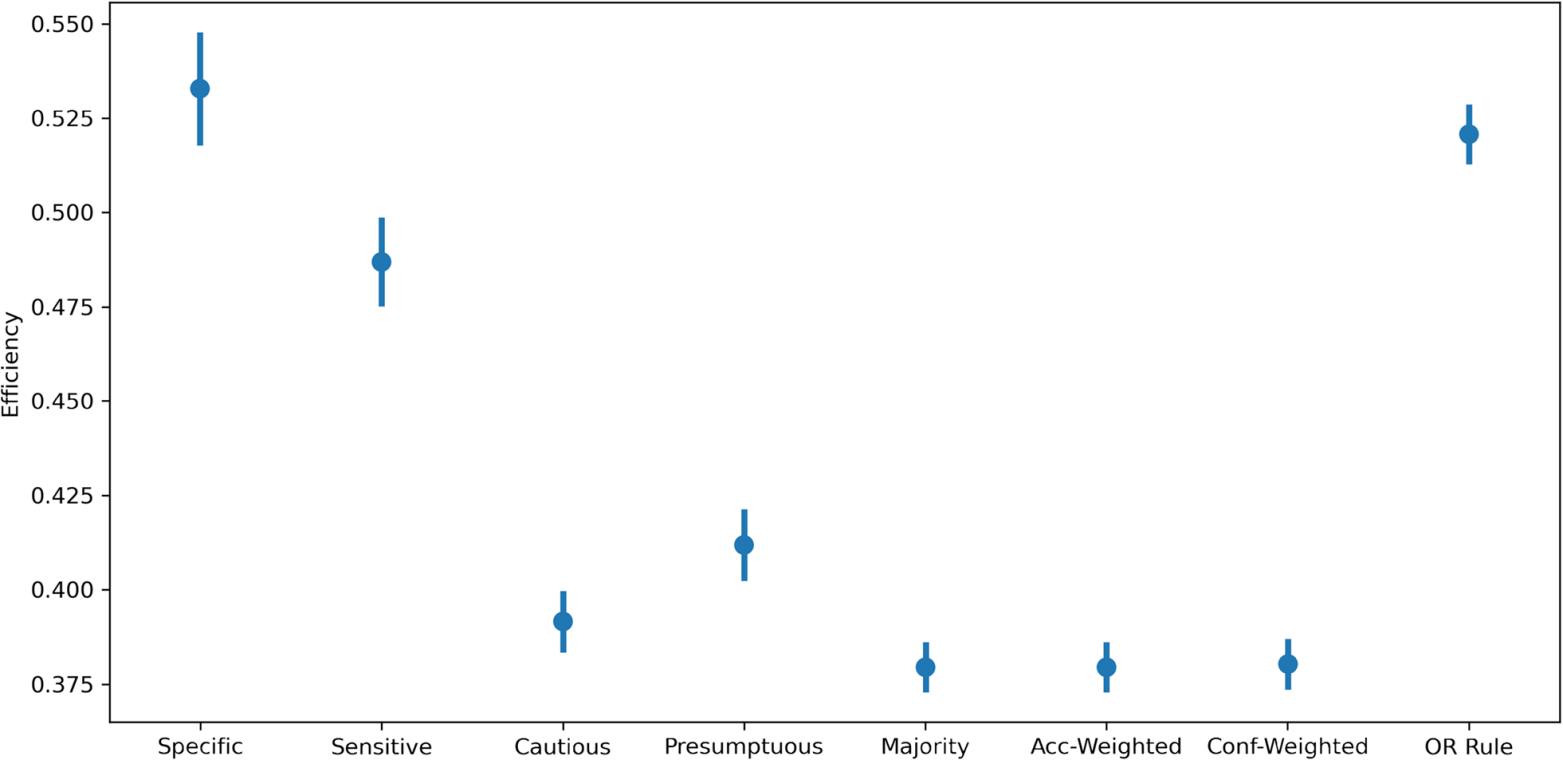
Average team efficiency and 95% confidence intervals for each of the 8 different protocols.

The results of the first experiment are depicted in Table 2, Figs. 5, 6, 7 and 8. The efficiency of the protocols is reported in Fig. 9.

**Table 2:** Results of the experiment regarding the first statement of the Kasparov’s Law: Average values of accuracy, sensitivity, specificity and $F_1$ score for both the Strong Machine and all the considered protocols. For each protocol and metric, we report both the average value of the metrics (across all 90 groups) and the (corrected) p-value arising from the comparison against the performance of the Strong Machine.

Protocol	Accuracy	Sensitivity	Specificity	$F_1$ score
Strong machine	84.54	94.76	76.27	84.52
Specific	85.05 ($p = 0.006$)	76.08 ($p < 0.001$)	92.32 ($p < 0.001$)	83.36 ($p < 0.001$)
Sensitive	83.38 ($p < 0.001$)	84.47 ($p < 0.001$)	82.50 ($p < 0.001$)	83.41 ($p < 0.001$)
Cautious	85.99 ($p < 0.001$)	79.88 ($p < 0.001$)	90.93 ($p < 0.001$)	85.01 ($p = 0.223$)
Presumptuous	82.37 ($p < 0.001$)	75.97 ($p < 0.001$)	87.54 ($p < 0.001$)	81.31 ($p < 0.001$)
Majority	87.66 ($p < 0.001$)	82.96 ($p < 0.001$)	91.47 ($p < 0.001$)	86.98 ($p < 0.001$)
Acc-Weighted	87.66 ($p < 0.001$)	82.96 ($p < 0.001$)	91.47 ($p < 0.001$)	86.98 ($p < 0.001$)
Conf-Weighted	87.75 ($p < 0.001$)	82.97 ($p < 0.001$)	91.62 ($p < 0.001$)	87.05 ($p < 0.001$)
OR Rule	81.29 ($p < 0.001$)	95.18 ($p = 0.033$)	70.04 ($p < 0.001$)	80.65 ($p < 0.001$)

In terms of accuracy, it can be seen from Fig. 5 and Table 2, that all three Majority-based protocols, the Cautious protocol and the Specific protocol reported a significantly higher accuracy than the Strong Machine. In terms of sensitivity, see Fig. 6 and Table 2, only the OR Rule reported a statistically significant superior performance compared with the Strong Machine. In terms of specificity, see Fig. 7 and Table 2, all protocols but the OR Rule reported a significantly higher performance. On the other hand, in terms of the harmonic mean of sensitivity and specificity, see Fig. 8 and Table 2, only the Majority-based protocols were significantly better than the Strong Machine, while the Cautious protocol was better only on average. Finally, we note that, despite the higher accuracy and predictive performance, all Majority-based protocols and the Cautious protocol were significantly less efficient than the other protocols (this is to be expected, as the former protocols always require at least two predictions to be elicited), while the Cautious protocol was still, on average, slightly more efficient than the Majority-based protocols.

### Second statement of the Kasparov’s Law

**Fig. 10:**
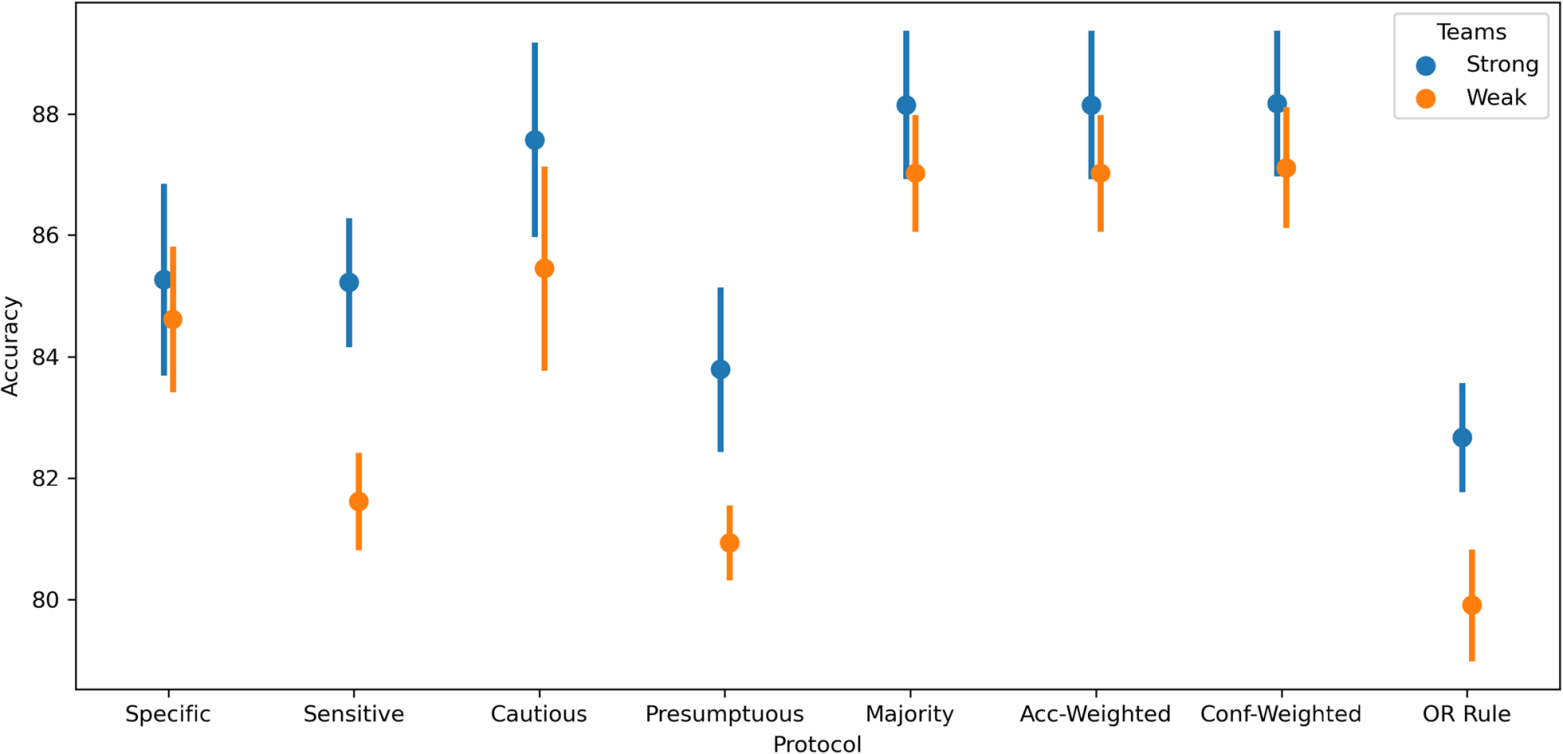
Average team accuracy and 95% confidence intervals for each of the 8 different protocols, for both Weak and Strong teams.

**Fig. 11:**
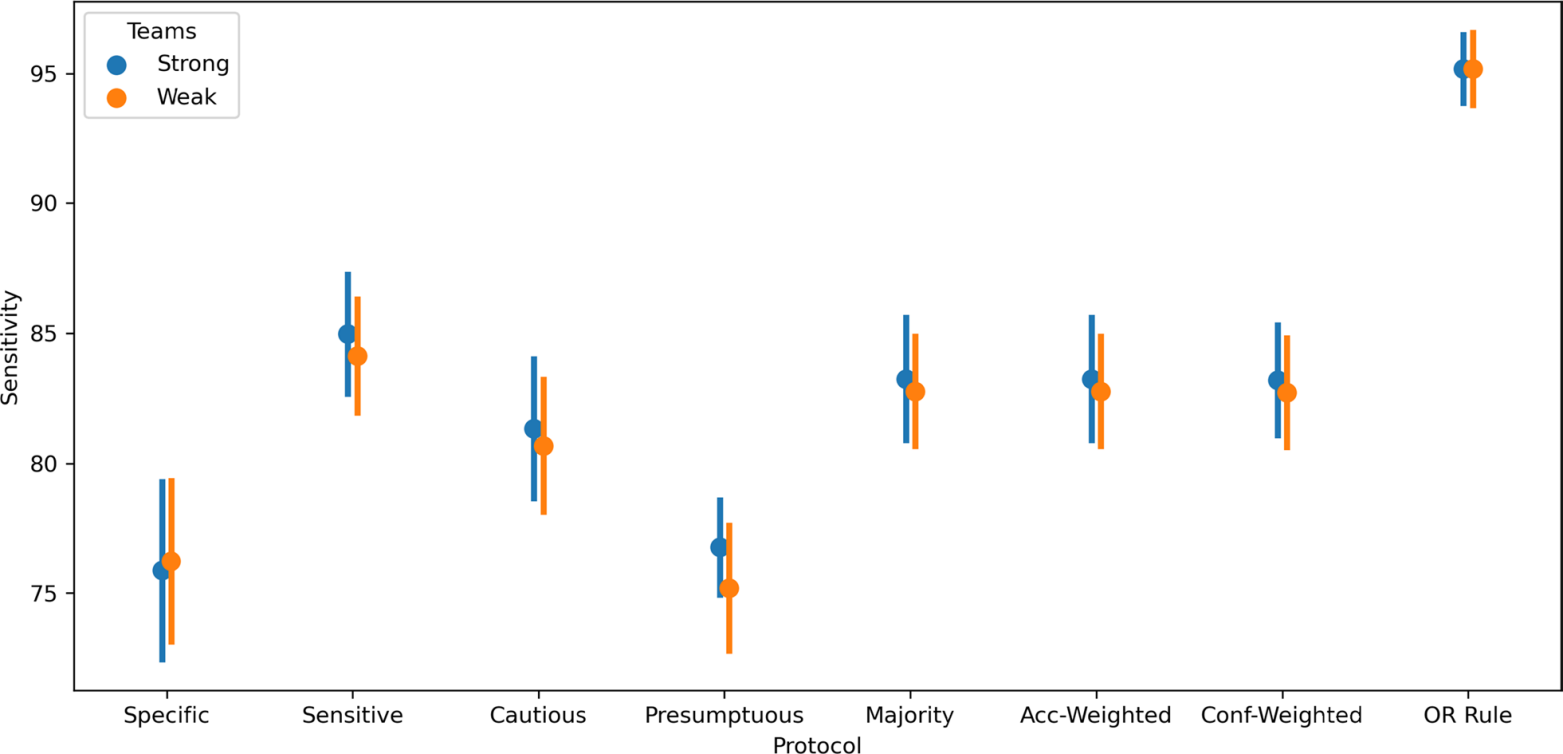
Average team sensitivity and 95% confidence intervals for each of the 8 different protocols, for both Weak and Strong teams.

**Fig. 12:**
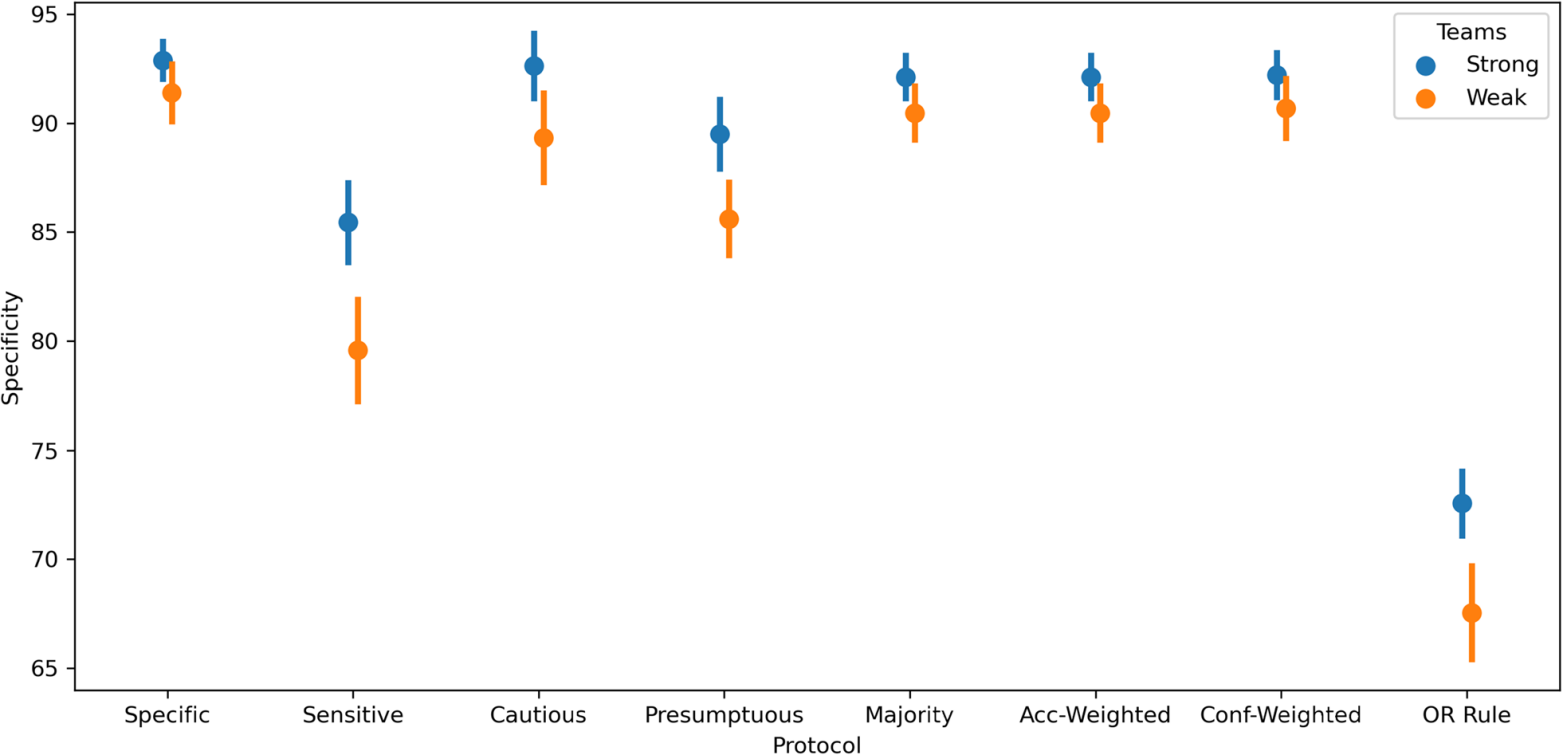
Average team specificity and 95% confidence intervals for each of the 8 different protocols, for both Weak and Strong teams.

**Fig. 13:**
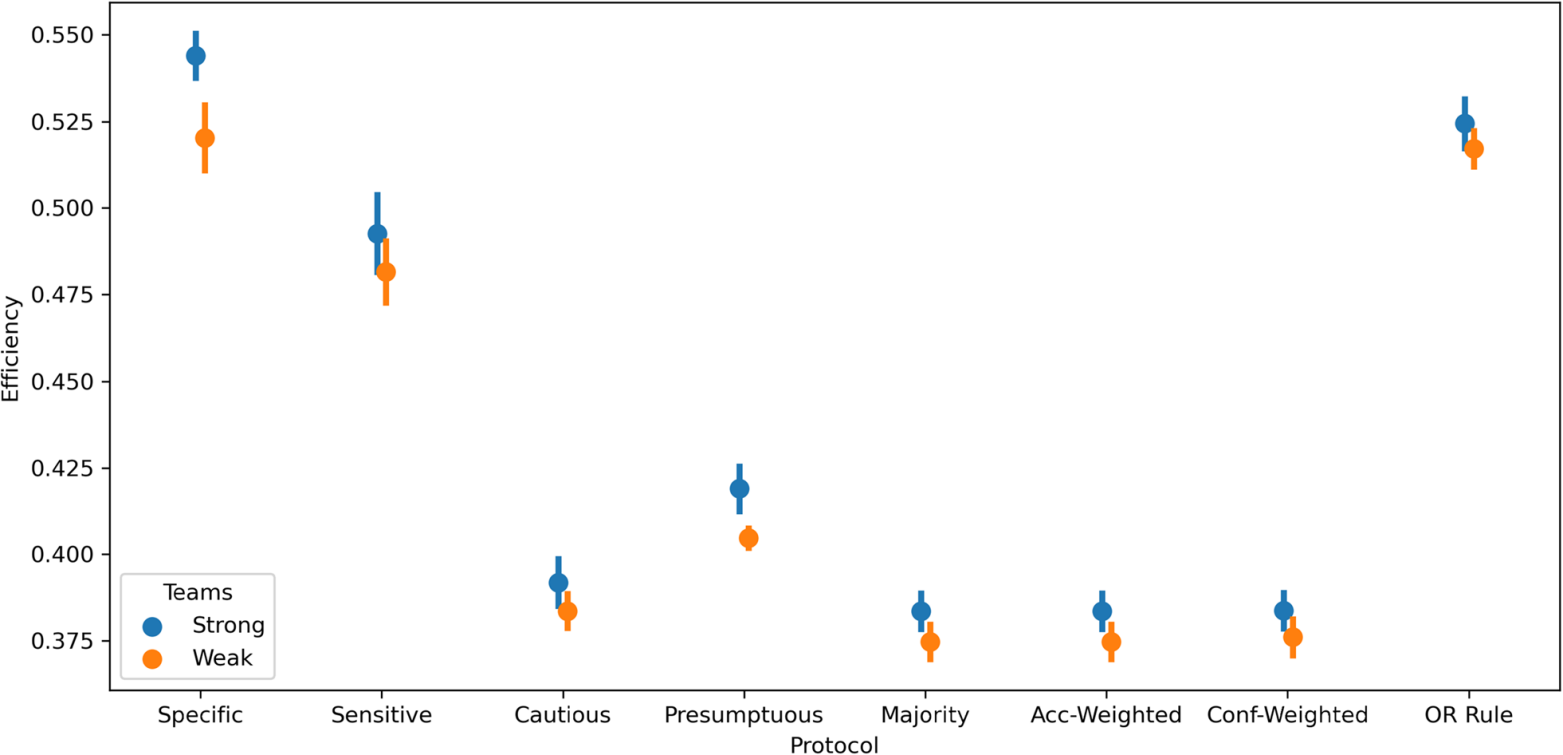
Average team efficiency and 95% confidence intervals for each of the 8 different protocols, for both Weak and Strong teams.

**Fig. 14:**
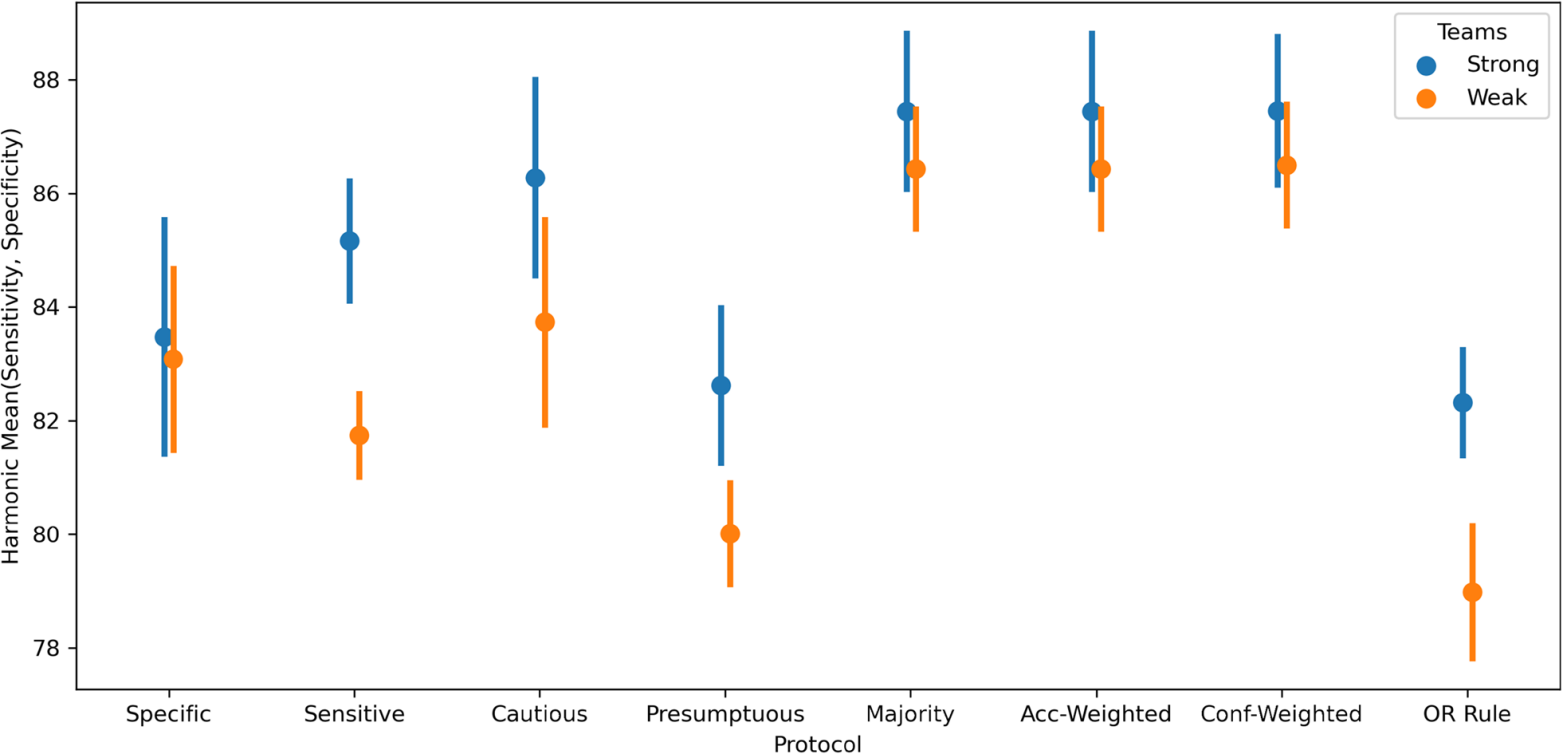
Average team value of the harmonic mean of sensitivity and specificity, along with 95% confidence intervals for each of the 8 different protocols, for both Weak and Strong teams.

**Fig. 15:**
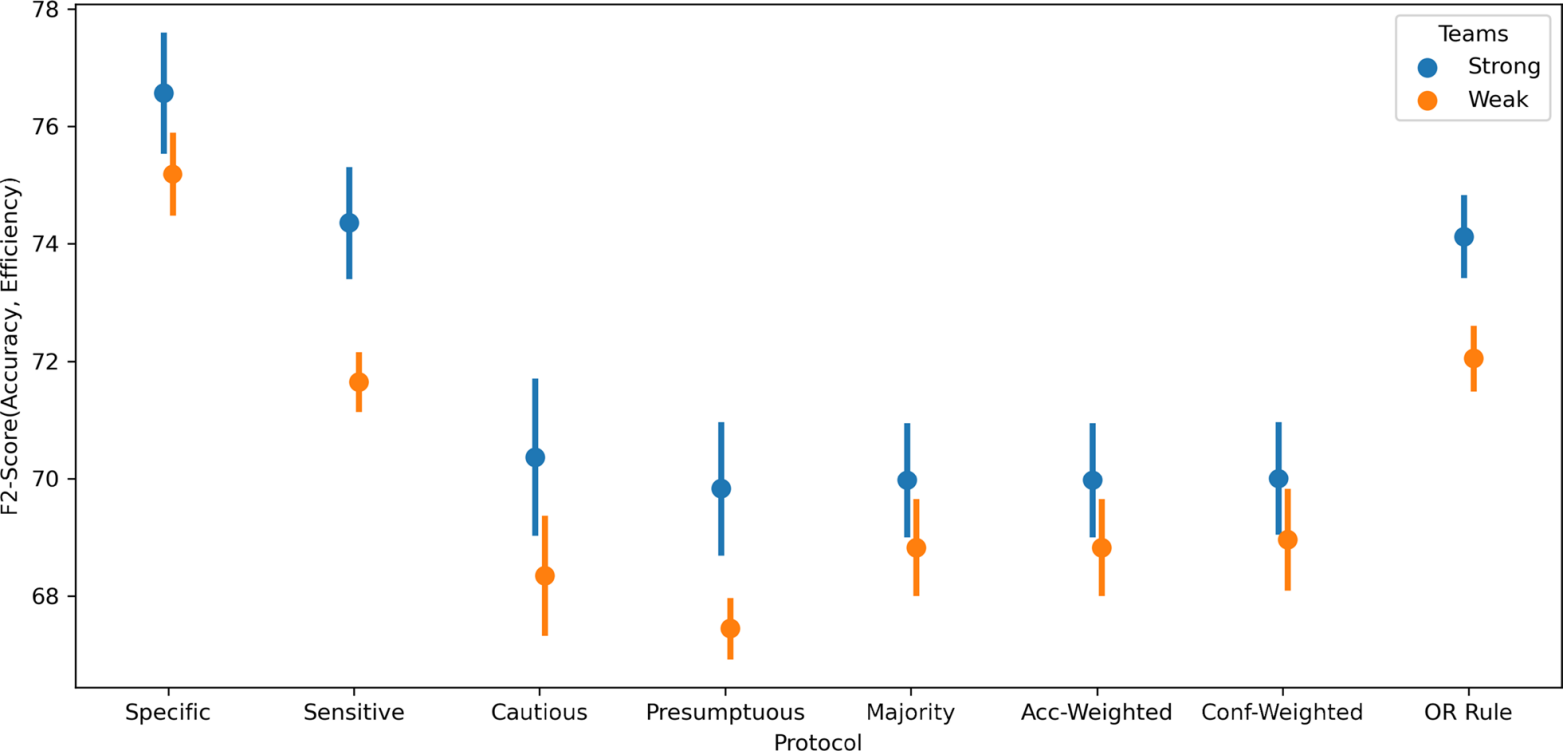
Average team value of the 2-order harmonic mean of accuracy and efficiency (denoted as F2-score in the y axis), along with 95% confidence intervals for each of the 8 different protocols, for both Weak and Strong teams.

The results of the second experiment are depicted in Tables 3, 4, 5, 6, 7, 8, 9 and Figs. 10, 11, 12, 13, 14 and 15.

**Table 3:** Results from the second experiment: for each metric and protocols, we report the performance of both the Strong and Weak teams.

Metrics	Teams	Specific	Sensitive	Cautious	Presumpt.	Majority	Acc	Conf	OR Rule
Accuracy	Strong	85.27	85.22	87.29	83.79	88.15	88.15	88.17	82.67
	Weak	84.61	81.62	84.61	80.94	87.03	87.03	87.12	79.91
Sensitivity	Strong	75.86	84.97	80.92	76.75	83.25	83.25	83.19	95.18
	Weak	76.23	84.14	78.98	75.18	82.77	82.77	82.72	95.18
Specificity	Strong	92.88	85.42	92.46	89.49	92.12	92.12	92.20	72.54
	Weak	91.40	79.58	89.17	85.59	90.47	90.47	90.68	67.54
$F_1$	Strong	83.47	85.16	86.27	82.62	87.44	87.44	87.45	82.32
	Weak	83.08	81.74	83.73	80.00	86.43	86.43	86.49	78.98
$F_2(acc, eff)$	Strong	76.57	74.36	70.37	69.83	69.98	69.98	70.00	74.12
	Weak	75.19	71.65	68.35	67.45	68.83	68.83	68.96	72.05
Efficiency	Strong	54.40	49.26	39.63	41.90	38.35	38.35	38.37	52.44
	Weak	52.04	48.15	38.65	40.47	37.48	37.48	37.61	51.72

**Table 4:** Corrected p-values for the pairwise comparisons w.r.t. accuracy in the Second Experiment

Strong/Weak	Specific	Sensitive	Cautious	Presumptuous	Majority	Acc	Conf	OR Rule
Specific	1.000	< 0.001	1.000	< 0.001	0.017	0.017	0.017	< 0.001
Sensitive	1.000	< 0.001	1.000	< 0.001	0.017	0.017	0.004	< 0.001
Cautious	0.004	< 0.001	0.017	< 0.001	1.000	1.000	1.000	< 0.001
Presumptuous	1.000	0.001	1.000	< 0.001	< 0.001	< 0.001	< 0.001	< 0.001
Majority	< 0.001	< 0.001	< 0.001	< 0.001	1.000	1.000	1.000	< 0.001
Acc	< 0.001	< 0.001	< 0.001	< 0.001	1.000	1.000	1.000	< 0.001
Conf	< 0.001	< 0.001	< 0.001	< 0.001	1.000	1.000	1.000	< 0.001
OR Rule	0.001	0.787	0.071	< 0.001	< 0.001	< 0.001	< 0.001	< 0.001

Strong teams are reported on the rows, while Weak teams are reported on the columns

**Table 5:** Corrected p-values for the pairwise comparisons w.r.t. sensitivity in the Second Experiment

Strong/Weak	Specific	Sensitive	Cautious	Presumptuous	Majority	Acc	Conf	OR Rule
Specific	1.000	< 0.001	1.000	1.0	< 0.001	< 0.001	< 0.001	< 0.001
Sensitive	< 0.001	1.000	0.001	< 0.001	1.0	1.0	1.0	< 0.001
Cautious	0.017	0.787	0.254	< 0.001	1.0	1.0	1.0	< 0.001
Presumptuous	1.000	< 0.001	1.000	1.0	< 0.001	< 0.001	< 0.001	< 0.001
Majority	< 0.001	1.000	0.001	< 0.001	1.0	1.0	1.0	< 0.001
Acc	< 0.001	1.000	0.001	< 0.001	1.0	1.0	1.0	< 0.001
Conf	< 0.001	1.000	0.001	< 0.001	1.0	1.0	1.0	< 0.001
OR Rule	< 0.001	< 0.001	< 0.001	< 0.001	< 0.001	< 0.001	< 0.001	1.0

Strong teams are reported on the rows, while Weak teams are reported on the columns

**Table 6:** Corrected p-values for the pairwise comparisons w.r.t. specificity in the Second Experiment

Strong/Weak	Specific	Sensitive	Cautious	Presumptuous	Majority	Acc	Conf	OR Rule
Specific	0.071	< 0.001	< 0.001	< 0.001	< 0.001	< 0.001	0.001	< 0.001
Sensitive	< 0.001	< 0.001	0.004	1.0	< 0.001	< 0.001	< 0.001	< 0.001
Cautious	1.000	< 0.001	0.017	< 0.001	0.254	0.254	0.254	< 0.001
Presumptuous	0.254	< 0.001	1.000	< 0.001	1.000	1.000	1.000	< 0.001
Majority	1.000	< 0.001	0.001	< 0.001	0.004	0.004	0.004	< 0.001
Acc	1.000	< 0.001	0.001	< 0.001	0.004	0.004	0.004	< 0.001
Conf	1.000	< 0.001	0.001	< 0.001	0.004	0.004	0.004	< 0.001
OR Rule	< 0.001	< 0.001	< 0.001	< 0.001	< 0.001	< 0.001	< 0.001	< 0.001

Strong teams are reported on the rows, while Weak teams are reported on the columns

**Table 7:** Corrected p-values for the pairwise comparisons w.r.t. efficiency in the Second Experiment

Strong/Weak	Specific	Sensitive	Cautious	Presumptuous	Majority	Acc	Conf	OR Rule
Specific	< 0.001	< 0.001	< 0.001	< 0.001	< 0.001	< 0.001	< 0.001	< 0.001
Sensitive	< 0.001	0.787	< 0.001	< 0.001	< 0.001	< 0.001	< 0.001	< 0.001
Cautious	< 0.001	< 0.001	0.017	0.017	< 0.001	< 0.001	< 0.001	< 0.001
Presumptuous	< 0.001	< 0.001	< 0.001	< 0.001	< 0.001	< 0.001	< 0.001	< 0.001
Majority	< 0.001	< 0.001	1.000	< 0.001	0.004	0.004	0.787	< 0.001
Acc	< 0.001	< 0.001	1.000	< 0.001	0.004	0.004	0.787	< 0.001
Conf	< 0.001	< 0.001	1.000	< 0.001	0.004	0.004	0.787	< 0.001
OR Rule	1.0	< 0.001	< 0.001	< 0.001	< 0.001	< 0.001	< 0.001	0.071

Strong teams are reported on the rows, while Weak teams are reported on the columns

**Table 8:** Corrected p-values for the pairwise comparisons w.r.t. $F_1$ score in the Second Experiment

Strong/Weak	Specific	Sensitive	Cautious	Presumptuous	Majority	Acc	Conf	OR Rule
Specific	1.000	0.017	1.000	< 0.001	0.004	0.004	0.004	< 0.001
Sensitive	0.004	< 0.001	0.254	< 0.001	0.254	0.254	0.254	< 0.001
Cautious	0.004	< 0.001	0.017	< 0.001	1.000	1.000	1.000	< 0.001
Presumptuous	1.000	1.000	1.000	< 0.001	< 0.001	< 0.001	< 0.001	< 0.001
Majority	< 0.001	< 0.001	< 0.001	< 0.001	1.000	1.000	1.000	< 0.001
Acc	< 0.001	< 0.001	< 0.001	< 0.001	1.000	1.000	1.000	< 0.001
Conf	< 0.001	< 0.001	< 0.001	< 0.001	1.000	1.000	1.000	< 0.001
OR Rule	1.000	1.000	1.000	< 0.001	< 0.001	< 0.001	< 0.001	< 0.001

Strong teams are reported on the rows, while Weak teams are reported on the columns

**Table 9:** Corrected p-values for the pairwise comparisons w.r.t. $F_2(acc, eff)$ score in the Second Experiment

Strong/Weak	Specific	Sensitive	Cautious	Presumptuous	Majority	Acc	Conf	OR Rule
Specific	0.004	< 0.001	< 0.001	< 0.001	< 0.001	< 0.001	< 0.001	< 0.001
Sensitive	1.000	< 0.001	< 0.001	< 0.001	< 0.001	< 0.001	< 0.001	< 0.001
Cautious	< 0.001	0.017	0.017	< 0.001	0.254	0.254	0.254	0.004
Presumptuous	< 0.001	< 0.001	0.071	< 0.001	0.787	0.787	1.000	< 0.001
Majority	< 0.001	< 0.001	0.004	< 0.001	1.000	1.000	1.000	< 0.001
Acc	< 0.001	< 0.001	0.004	< 0.001	1.000	1.000	1.000	< 0.001
Conf	< 0.001	< 0.001	0.004	< 0.001	0.787	0.787	1.000	< 0.001
OR Rule	0.071	< 0.001	< 0.001	< 0.001	< 0.001	< 0.001	< 0.001	< 0.001

Strong teams are reported on the rows, while Weak teams are reported on the columns

In terms of accuracy, see Fig. 10 and Table 4, the teams of weak observers exhibited higher performance than the teams of strong observers when any of the Majority-based protocols was applied, except for the Cautious and Majority-based protocols: notably, the difference was statistically significant with respect to the Sensitive, Specific, Presumptuous and OR Rule protocols. Moreover, for all the Majority-based and Specific protocols the difference between the teams of weak and strong observers was not statistically significant. In regard to sensitivity, see Fig. 11 and Table 5, the OR Rule protocol (for both the weak and strong teams) reported a significantly higher performance than all other protocols, as expected; moreover, in all cases, the difference in performance between the teams of weak and strong observers was not statistically significant. Notably, the sensitive protocol and all Majority-based protocols applied to weak observers were significantly better than the specific and presumptuous protocols applied to strong observers, and slightly so for the cautious protocol.

In regard to specificity, see Fig. 12 and Table 6, the Specific protocol for the teams of weak observers reported a performance which was comparable with, or statistically significantly better than (for the Sensitive and OR Rule protocols) all the strong teams protocols.

Finally, in regard to efficiency, see Fig. 13 and Table 7, the Specific, Sensitive and OR Rule protocols (for both weak and strong teams) reported a significantly better performance than all other protocols. Notably, for the OR Rule, Sensitive and Confidence-Weighted protocols the difference between strong and weak teams was not statistically significant. Moreover, the Specific, Sensitive and OR-rule protocols applied to weak observers were significantly more efficient than the other protocols applied to strong observers.

## Discussion

The essence of cooperative work is “the interdependence of multiple actors [possibly engaged in separated activities or tasks] who interact through changing the state of a common field of work” [41] to produce some common good or service. In our case, the “field of work” can be imagined as a “box” where each image reader puts their decision token (and hence the respective data structure). This interdependence is usually managed by a *coordinative protocol* that is “an integrated set of procedures and conventions stipulating the articulation of interdependent distributed activities”, like e.g., making a decision on a MRI scan. Coordinative protocols are a kind of interaction protocol stipulating how multiple actors (pulled together to form a collective) influence each other so to have some work (and product) done. In this study, we have investigated a number of interaction (coordinative) protocols by which the service of “reporting radiological findings” (which can inform subsequent action and services, like a specific health intervention or treatment) can be produced, and that do not require direct communication among the actors involved. These protocols are all variations of the main setting of *double reading* radiological imaging, which is different from *dual reading* in that this latter setting involves two or more readers who communicate and discuss to *collaboratively* produce the report, often as members of the same team. In both cases, we can frame these protocols as structured ways to leverage the *collective intelligence* of an ensemble of image readers, that is to gain a higher decision accuracy than involving each reader individually (at the expense of some efficiency, but not necessarily so), avoiding some pitfalls of direct interaction where “the more” is not always “the better”, mainly due to bandwagon effect, priming and truth bias [25, 34, 43].

In fact, in all the protocols described in Sect. 2, the first two readings and interpretations are produced independently of each other, and the second is always given by a medical AI: this is justified to minimize automation bias [31, 44] in light of several researches finding that the machine advice either does not improve the interpretation of expert readers [29] or can even mislead them in their judgments, for instance in screening tasks (e.g. [3]) and those based on the visual inspection of diagnostic images [14]; this latter phenomenon can occur not necessarily only because doctors over-rely on the machine and over-trust its advice, but also for the opposite reason, because they have a negative prejudice against it [6] and are negatively “primed” by its advice.

As anticipated in the Introduction, our investigation allows to contrast the performance levels evaluated for different interaction protocols and the intuitive notions evocatively expressed in terms of the so called Kasparov’s law, which is based on informal observations in the realm of recreational team work, namely freestyle chess. As said above, we are not interested in an empirical proof of this intuition as such, but in discussing what in that formulation (see Sect. 1) is denoted as superior (and inferior) process. In fact, what makes a process good, or better than others, depends on contextual factors, and on the relevant dimensions of interest. Moreover, not all these dimensions are equally measurable, regardless their relevance in human collaborative settings. Thus, we decided to focus on accuracy, broadly meant in terms of complement of error rate^4^; and on efficiency, for the immediate value of these concepts in any practice, especially those where errors can result in harm to people and where long-term sustainability is priority. In what follows, we will comment the results reported in Sect. 3 along these two dimensions.

**Fig. 16:**
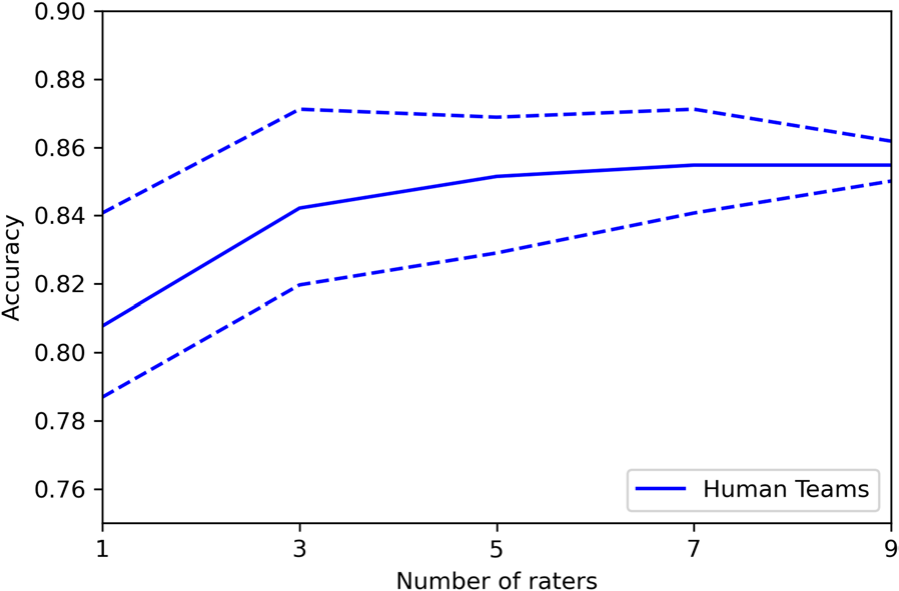
Average, minimum and maximum team accuracy, with respect to groups of varying number of observers (from 1 observer, to 9 observers).

Let us consider the first “part” of the Kasparov’s Law first: this could be rephrased in terms of *more weaker agents + superior process > one stronger agent + inferior or no process*, where a superior process distinguishes itself from an inferior one in its ways to positively combine the contributions of multiple agents. Although there are many ways to combine different judgements together (we explored some methods applied to ensemble machine learning in [10]), majority voting has been found quite effective in many fields and domain since time immemorial [11]. We could call this observation the Aesop’s Law, as one of the famous fables of this writer from the 6th century BCE (namely the no. 53, The Bundle of Sticks) inspired countless variations of the old saying running “union is strength” (or “in unity is strength”). Indeed in our study, the protocols involving 3 readers and majority voting, and hence a decision best of three, are easily those associated with the more accurate group performance, in accordance with other recent studies [48]. In Fig. 16, we depict the performance of groups of varying number of readers (from our sample) to which the simple majority protocol is applied: we can see how involving more than one rater in each diagnostic decision yields more accurate decisions than leveraging the judgment of individuals. It is also worthy of note that involving more than 5 readers is not associated with a relevant increase in accuracy; in fact, the greater increase in accuracy occurs when moving from single decisions to “best-of-three” decisions and, therefore, to involve more readers would likely result in a waste of resources.^5^

We can see from Figs. 5 through 12 that, in all cases, given a specific metric, the best performing protocol was always a protocol that could be considered as “fit for use” for that metric, indeed:

- If our goal is to maximize classification performance in the average case then we focus on accuracy: in this case, the best performing protocols were the Cautious protocol and the Majority-based ones. Indeed the Majority protocol is designed to maximize accuracy, as any disagreement among the first two observers would lead to the involvement of a third observer: thus, the probability of error for this protocol (assuming independence of the observers) scales approximately as $err^3<< err$ (where *err* is the error rate of the worst observer in the team). On the other hand, the Cautious protocol is aimed at offering an acceptable trade-off between accuracy and efficiency, on the basis of the parameters $\tau$ and $\delta$, so to interpolate between the Majority and Presumptuous protocols;
- If our goal is to correctly identify as many abnormal cases as possible, then we focus on sensitivity: in this case, the best performing protocol was the OR Rule, followed by the Sensitive protocol. Indeed, in the OR Rule it suffices for one observer to identify the case as abnormal; by contrast, the Sensitive protocol attaches more importance to the classification of the first observer and, thus, is more conservative than the OR Rule (consequently, this is reflected by a significantly higher specificity);
- Finally, if our goal is to correctly identify as many normal cases as possible, then we focus on specificity: in this case the best performing protocol was the Specific one, as expected. We note, also, that the Cautious and Majority-based protocols reported a specificity comparable to that of the Specific protocol, but with a much worse efficiency.

In our study, the Kasparov’s Law is recognized to hold in several cases, highlighted in Sect. 3. Here we emphasize the case of the cautious protocol, which makes wise use of the additional information collected from the readers, the perceived complexity of the case and their confidence in their classification: this protocol, which is also significantly more efficient than the majority voting schemas (see Fig. 9; Tables 2 and 3), significantly outperformed the strong machine, which – we recall – was more accurate than any of the readers involved. If, nevertheless, sensitivity is the target quality to optimize, we see that a group of just 2 raters can perform equally (or slightly better) than a super-human AI (see the OR-rule protocol in Fig. 6). Likewise, the humans are significantly more accurate than the stronger machine for all the collaborative protocols, but one (see Fig. 7). This suggests that, depending on the readers involved and on the quality dimension along which we want to optimize the overall performance, investing on protocols of human-human collaboration can be more cost-effective than procuring a state-of-the-art AI system. This complements the findings reported in [1], where also investing on human resources and a specific perceptual training was found to be associated with better performances than those of the best AI system. Moreover, in regard to the accuracy-efficiency balance the cautious protocol achieved an average higher score than all the majority voting protocols (which was not expected), although not significantly so. To this respect, the specific and sensitive protocols (including the OR-rule one) significantly outperformed the other protocols, even when only weak observers were involved.

### Limitations and future work

As first objection to what discussed above, it could be observed that the detection of knee lesions is not a typical task for which double reading is usually applied. In specialist diagnosis, *dual reading* has been so far preferred, that is the simultaneous reading by two observers to reach a consensus. On the other hand, double reading usually finds justification for the screening of large numbers of healthy individuals for diseases with an important social cost and individual burden (such as breast cancer) and not for conditions for which a missed or wrong diagnosis has a relatively low impact (as in the case of many post-traumatic or degenerative orthopedic conditions). However, the application of this protocol also to triage and any specialist diagnostic task is more a matter of finding a balance between the need to curb resource consumption (above all the involvement of multiple readers) and the meritorious goal to reduce the rate of diagnostic error and increase patient safety, regardless of the health problem. Although this resource-intensive protocol has been so far considered only for selected, high-risk examinations, our point is that the availability of accurate and reliable medical AI could change this state of affairs, especially if the increasingly wider diffusion of online AI-based services, provided in SaaS (Software as a Service) will reduce the cost of the AI recommendation, at the level of single transaction. This would make the use of AI in double-, dual- and over-reading tasks competitive with respect to the involvement of human second readers and arbiters.^6^

Speaking of costs suggests that we also address another possible limitation of this study. We are aware that a comparison between medical protocols can, and should, also be conducted in terms of cost-effectiveness, for example by calculating the incremental cost-effectiveness ratio (ICER), i.e. the expected cost per one additionally detected pathological condition [35] or, even better, in terms of quality adjusted life years (QALYs). Nevertheless, these studies are strongly dependent on the type of examination, modality, setting, impact of the disease and its prevalence; doing so for knee MRI would have made our study lose generality, which instead focuses on the comparison of different ways of integrating a technological support in double-reading protocols in general.

Third: two protocols out of eight require additional information to be collected from the observers in their image reading task: namely, the complexity of the case (as this is perceived by the observers), and the confidence on their judgment for that case. We acknowledge that doing so entails an additional effort for the observers and the need to set further data structures in the reporting platform. Furthermore, we reported how the protocols using this additional data were not particularly better than the others in this study. However, we observe that collecting these data is a low-impact requirement, all the lower the more knowledge can be extracted to understand “who is right” among the observers (including the AI) and hence make a better decision at the best of two judgments. Then, we acknowledge that we did not fine-tune the above protocols to fully exploit the additional information coming from knowing the case complexity, the observer confidence and their accuracy; however, the cautious protocol is almost as accurate as the majority ones (see Figs. 5, 10 and 14; Tables 2 and 3) but much more efficient (see Figs. 9, and 13) and slightly preferable taking into account both dimensions (see Fig. 15 and Tables 3, and 9). That said, we doubt that a general way to combine the above data together to maximize effectiveness or efficiency in *all of the* decision settings exists: their combination depends on the distributions of those attributes and the correlations among each other (e.g., see Fig. 2c). That notwithstanding, we deem two directions worthy of further investigation: case complexity could be evaluated on a probabilistic scale (i.e., “how likely it is that an expert radiologist could get this case right?”), so that promising meta-cognition techniques, like the “surprisingly popular” method [36], for aggregating decisions across a group of people could be applied. Also, the reader accuracy, instead of being evaluated on all the available decisions made by that reader (as we do in the accuracy-weighted majority protocol), could be evaluated on the most similar cases to the one at hand, once the representativeness of this point with respect to the available data has been verified (e.g., through the techniques presented in [8]).

Lastly, this study is limited to investigating cooperative protocols where the direct interaction and communication between humans and the AI is purposely excluded: future work should also be aimed at shedding light on the potential differences in medical performance arising from protocols where humans consult the AI advice, provide a feedback about it (cf. active learning) and explore the reasons behind it [23], both in individual settings (1-to-1) or in collaborative ones (many-to-1), like in [40]. In these studies, other dimensions than accuracy and efficiency should be considered to evaluate the protocols, like: user satisfaction, AI acceptability, user trust and human sustainability: in particular, considering the latter aspect entails to consider the extent the prolonged use of the AI support would induce some form of deskilling [9], opportunistic practices [45] and automation biases [31] in their users.

## Conclusions

Human-AI collaboration is an important area to invest further research on, especially in medicine, and radiology more in particular, since finding efficient ways to combine the advice by humans and intelligent machines in double-reading tasks can improve report accuracy, help to address the radiology workforce crisis [18], and, recently, provide a solution for dealing with the long-term backlog due to COVID-19 [12].

Among those who are interested in human-AI collaboration, the famous remark by Garry Kasparov known as the Kasparov’s law is a sort of adage and yet a still-to-prove conjecture: in this paper, we have focused on this conjecture, not only to provide a first informal (and yet statistically significant) confirmation of it but, above all, to emphasize the importance of properly designing the interaction protocols by which humans and machines can cooperate. Indeed, we have observed that good interaction protocols guarantee better decision performance that easily surpass the performance of individual agents, even of realistic super-human AI systems. In this respect, the main challenges lie in designing protocols that are balanced in terms of accuracy and efficiency, i.e., viability. Moreover, focusing on how humans and AI can collaborate, rather than on evaluating their performance as single and isolated agents, would also allow to go beyond the studies that report improbable comparisons (e.g. [20, 33]) or those that promote small improvements of algorithms over state-of-the-art solutions or even traditional methods (like linear regression [19]), but often neglect the much deeper issue of the poor reproducibility of results [13]

To contribute to this new body of works, we have discussed the case in which humans who perform worse than a powerful AI (even by several percent points) can outperform it if their judgments are aggregated by majority voting in double-reading settings: this “proves” the first part of the Kasparov’s law but it is also an informal confirmation of the much older Aesop’s law, i.e., union is strength. This connects with recent studies about how the collective intelligence of several relatively weak decision makers can equate, or even surpass, the performance of a (much more expensive) super-expert doctor (e.g., [4]).

We also showed how small ensembles of significantly weaker (i.e., less accurate) MRI readers (including an average AI) can significantly outperform equally numerous teams of stronger (i.e., more accurate) readers, supported by the same computational tool, when the former ones are engaged in some “better” fit-for-use interaction protocols (where better can be interpreted in different ways, according to some requirement of accuracy / efficiency trade-off). This is also compatible with the second part of the Kasparov’s law: nevertheless we would propose a wider interpretation of these findings in terms of two succinct conjectures of general scope: first, safer and more human sustainable care practices can be achieved by focusing more on how to guarantee a better cooperation within hybrid humans-AI teams (and hence on the design of apt interaction protocols that are optimized for some dimension of interest), than investing only on the technological component of the above teams. Nevertheless, if a hospital management still makes the decision to introduce a medical AI into a team of doctors, this should be at least as accurate as the average doctor (duly evaluated, as we advocated in [7]), or better than that but not worse, unless a proper interaction protocol is designed and adopted to leverage the best capabilities from both doctors and machines.

## Data Availability

The annotation data that support the findings regarding the machine learning experiment are property of the IRCCS Orthopedic Institute Galeazzi. Data can be shared upon reasonable request and with permission of the above Institute.

## Footnotes

1 That notwithstanding, the effect of this innovation on waiting lists would be more uncertain as it depends also on how such an efficiency increase will reinforce the so-called phenomenon of biomarkup [32], that is the tendency in relying on an increasing number of biomarkers for diagnostic and prognostic purposes leading to an excess of imaging and laboratory tests.

2 We recall that the total number of 2-permutations of n objects is $n(n-1)$.

3 This is an average measure where the effectiveness of a medical decision is considered 2 times as much important as efficiency, for the importance to positively contribute to someone’s health and well-being.

4 In doing so we also considered the kind of error, false positive or false negative, hence focusing on specificity and sensitivity, respectively

5 This is true when all the observers are similar in their skills, as it was our case.

6 For instance, Zebra Medical Vision, one of the few vendors of medical AI offering diagnostic services for musculoskeletal conditions, allows healthcare providers to access their algorithms for 1 USD per scan (see https://www.zebra-med.com/solutions). In contrast, in Italy the costs related to radiological reporting amount to approximately one fifth of the reimbursed amount, that is approximately 30 euros for knee MRI (2020 fees).
